# Patient Satisfaction with Implant-Supported Monolithic and Partially Veneered Zirconia Restorations

**DOI:** 10.1155/2021/6692939

**Published:** 2021-02-06

**Authors:** Paolo De Angelis, Giulio Gasparini, Edoardo Rella, Silvio De Angelis, Cristina Grippaudo, Antonio D'Addona, Paolo Francesco Manicone

**Affiliations:** ^1^Department of Head and Neck, Division of Oral Surgery and Implantology, Institute of Clinical Dentistry, Fondazione Policlinico Universitario A. Gemelli IRCCS—Università Cattolica del Sacro Cuore, Rome, Italy; ^2^Department of Head and Neck, Division of Oral and Maxillofacial Surgery, Institute of Clinical Dentistry, Fondazione Policlinico Universitario A. Gemelli IRCCS—Università Cattolica del Sacro Cuore, Rome, Italy; ^3^Private Dental Practice, Ascoli Piceno, Italy

## Abstract

The digital workflow and the application of Computer-Aided Manufacturing (CAM) to prosthodontics present the clinician with the possibility of adopting new materials that confer several advantages. Especially in the case of zirconia, these innovations have profoundly changed daily practice. This paper compares the satisfaction and perception of patients who received implant-supported single crowns (SC) and fixed partial dentures (FPD) made from zirconia, either monolithic or partially veneered, after 3 years of follow-up; the success and survival rate of these restorations were also measured. Forty patients, who had been previously treated with implant-supported SC or FPD, either monolithic or partially veneered, and submitted to a yearly maintenance program, were recalled 3 years after their treatment and requested to complete an 8-question questionnaire regarding their perceptions of the treatment. Any mechanical or biological complication that had occurred from the time of delivery was also recorded. Patients that experienced ≥1 complication were less likely to be prone to repeat the treatment. The 3-year success rate was 92.6% for monolithic restoration and 92.3% for partially veneered restoration, while the survival rate was 100% for both restorations. The 3-year follow-up found that monolithic and partially veneered zirconia restorations are both well-accepted treatment options, and patients preferred the veneered restorations (0.76, *p* < 0.05) from an aesthetic point of view. According to our results, monolithic and veneered zirconia restorations are both reliable treatment options and are both equally accepted by patients.

## 1. Introduction

The rapid evolution of CAD/CAM (Computer-Aided Design/Computer-Aided Manufacturing), and the advancements of its application to dentistry have heralded a series of innovations in all branches, especially in implantology and restorative dentistry, where its association with new materials presents the clinician a new treatment possibility that is both economically advantageous and clinically resilient [1–3].

Two of the most commonly used materials in fixed prosthodontics, zirconia (ZrO_2_) and lithium disilicate, are commonly utilized in digital workflows; while zirconia can only be used with this technology, several reports agree that pressed lithium disilicate nevertheless produces better clinical performances [4, 5].

The adoption of CAD/CAM in implant dentistry can provide the clinician with abutments, both in zirconia or titanium, that are shaped appropriately to the position of the implant and the soft tissue characteristics.

Among these materials that can be milled with CAD/CAM technology, zirconia, the crystalline dioxide of zirconium, is by far the most adopted; thanks to its mechanical properties and aesthetic capabilities, it has been also termed ceramic steel [6–8].

Zirconia exists in monoclinic, tetragonal, and cubic phases; with the addition of stabilizing oxides such as MgO, CaO, or Y_2_O_3_, first- and second-generation zirconia are frozen in the tetragonal condition, preventing the so-called martensitic transformation [6]; instead, third-generation zirconia is metastable in the cubic phase [9].

First-generation, or conventional, zirconia, developed almost 20 years ago, has a high light refraction index; therefore, it is an extremely opaque material. As a consequence of these compromised aesthetic characteristics, conventional zirconia is used as a substitute for the cast metal core and therefore veneered with glass-ceramic, providing higher translucency and overall better aesthetic [10]. The adhesion between the veneer and the core has improved since the introduction of this material; nevertheless, cohesive fracture, where a thin layer of ceramic remains on the framework, is still a common complication [11].

To prevent this, zirconia can be used in monolithic restorations, where the whole crown is made of zirconia and no veneers are used. Certain requirements must be met before this material can be used in a monolithic fashion: it is critical that the material is sufficiently translucent and aesthetically pleasing: these requirements are especially met with second-generation zirconia (3Y-TZP), where the number and grain size of aluminum oxide are reduced in terms of number and dimension and repositioned in the zirconia framework: this allows for higher transmittance of light, with good stability and high strength, even if lower than the previous generations of zirconia [12].

To achieve the translucency of other glass-ceramics, third-generation zirconia (5Y-TZP) was introduced; contrary to the previous two generations, this zirconia contains up to 53% of the cubic phase: this was achieved by the introduction of a higher percentage (from 4% to 5%) of yttrium. Third-generation zirconia has quite interesting properties: it can be used at extremely low occlusal thickness [13] thanks to its higher flexural strength; therefore, it is more conservative than other conventional restorative materials [14] (such as lithium disilicate or porcelain fused to metal) while at the same time providing the appropriate aesthetics, [15] especially when layered precolored zirconia is used, which offers several aesthetic advantages, and can help manufacture a more natural and aesthetically pleasing crown when compared to monochrome zirconia [16].

The main purpose of this paper was to analyze, via a questionnaire, the satisfaction and perception of patients who received a monolithic or partially veneered implant-supported restoration, either a single crown (SC) or a fixed partial denture (FPD), at 3 years after delivery. Also, the restorations were analyzed at the 3-year follow-up, and the antagonist was inspected to evidence any wear of its occlusal surface. The clinical outcomes after the 3-year follow-up, e.g., the frequency and type of complications, were also recorded.

## 2. Materials and Methods

### 2.1. Study Design

Patients who had undergone monolithic or partially veneered zirconia SCs and FPDs, in the molar to premolar area, on dental implants between January 1, 2017, and June 1, 2017, were screened and invited to participate in the survey.

All procedures took place at two private dental practices in Ascoli Piceno, Italy, and Rome, Italy. All procedures were performed according to the Declaration of Helsinki guidelines on experimentation involving human subjects. Each participant enrolled in the study received adequate explanations on the study design and objectives and provided written informed consent. Due to the retrospective nature of this study, it was granted an exemption in writing by the local ethics committee.

### 2.2. Participants

We included patients that (1) were willing to provide informed consent and participate in the study and (2) had available information on the date the prosthesis was delivered and on the eventual complications. We excluded patients that (1) could not answer to the questionnaire due to neurological or psychological disorders.

### 2.3. Clinical Procedures

The implant position was based on a prosthetic-guided planning developed after performing an exhaustive clinical and radiologic examination; implant fixtures were inserted under local anesthesia following the manufacturer's guidelines (Straumann Implant System, Biomet 3i Implant System).

After a healing period of 3 months, either a conventional impression or a digital impression was taken.

In subjects that followed a conventional workflow, an impression was taken with alginate impression material (Xantalgin, Mitsui Chemical Group, Tokyo, Japan) and stock trays to manufacture a custom impression tray. The final impression was taken using the open tray technique and polyether impression material (Impregum Penta Soft Quick Step MB, 3M ESPE) following the manufacturer's guidelines.

In subjects that followed a digital workflow, a scan-body abutment was screwed to the implant body, and a scan of both arches, as well as of the occlusion, was registered (Trios 3, 3SHAPE). The standard tessellation language (STL) file was then sent to the dental technician.

The restorations were designed as either cement-retained or screw-retained. If feldspathic porcelain was to be added on the buccal side, the zirconia structure was intraorally tried with the buccal cut back applied and then sent back to the dental laboratory to be veneered.

High-translucency (HT) zirconia was utilized, in the form of inCoris TZI (Sirona) and Biodynamic Multilayer 1200/600 Mpa Progressive (Biodynamic). The former is a HT zirconia with flexural strength > 900 MPa, while the latter is a more innovative material that presents higher flexural strength in the cervical region (1200 MPa), where more mechanical strength is needed, and lower flexural strength (600 MPa) in the incisal region, where more translucency is preferred.

Cement-retained prostheses were cemented with a glass-ionomer cement on titanium stock abutments, previously screwed to the implant fixture following the manufacturer's guidelines, carefully removing the excess cement. FPDs (hybrid cement-screw-retained) were bonded extraorally to prefabricated metal substructures (screw-retained abutments). Screw-retained single crowns were bonded to prefabricated titanium base abutments. All the crowns and bridges were bonded using an adhesive luting composite (Multilink Hybrid Abutment, Ivoclar, Schaan, Liechtenstein) and finally polished.

The screw-retained prostheses were seated on the implants and screwed using a manual torque control ratchet (20 N/cm). The screw access holes were packed with polytetrafluoroethylene tape and covered with composite resin. The complete removal of excess cement and seating of the restorations were checked with a radiograph taken immediately after delivery of the restoration.

After the prosthesis delivery, all patients were enrolled in a personalized maintenance care program based on the risk assessment of the patients.

### 2.4. Outcomes and Data Collection

The primary purpose was evaluating if, 3 years after the rehabilitation, patients were satisfied from a functional and aesthetic point of view, and if they were willing to undergo the same procedure in the future, if needed. The secondary purpose was to define clinical outcomes, such as the number and type of complications, and wear of the restoration and of the opposing dentition after a 3-year follow-up.

A restoration was defined as a success if there had not been any kind of complication; a restoration was defined as surviving if it was still in use at the 3-year follow-up [17].

The analyzed factors were the use of axial or tilted implants, the type of edentulism, the presence or absence of parafunctions, and the design of the restoration (either screw-retained or cement-retained and either monolithic or partially stratified).

Furthermore, we examined the correlation between the frequency of mechanical and biological complications and the willingness to undergo a similar procedure.

The following information was obtained from the clinical chart:

- Number and type of implants placed
- Type of rehabilitation
- Presence of parafunctions
- Number and type of complications

Each recruited subject contributed with a single rehabilitation.

At the recall visit, the corresponding restorations and opposing dentition were examined.

Complications were divided into technical and biological. Technical complications were also divided into major and minor complications, as suggested by Lang et al. [18].

Wear of the restoration and/or the opposing dentition were clinically assessed using magnifying loupes.

Biological complications were assessed by performing periodontal probing and recording the probing depth (PD), bleeding on probing (BoP), and presence of suppuration. Marginal bone loss was analyzed using standardized intraoral radiographs at baseline, after prosthesis delivery, and at the 3-year follow-up. Peri-implant mucositis and peri-implantitis were defined following the guidelines by Renvert et al. [19].

An independent investigator provided subjects with a questionnaire, which started with a question asking them to indicate the location of the rehabilitation. Data for the analyses were extracted only from the questionnaire of subjects who had provided the correct answer to the initial question. An operator external to the previous treatment was instructed to collect these questionnaires [20].

The patients were also asked to mark on 100 mm visual analogue scales (VAS) the appropriate answers to the following questions:

1. How would you rate the appearance of your teeth immediately after their treatment?
2. How would you rate the appearance of those teeth today?
3. How would you rate your present capacity to chew?
4. How would you rate your present capacity to speak?
5. How easy do you find it to clean your teeth and gums?
6. What did you think about the financial cost of your treatment at the time of treatment?
7. In hindsight, how would you rate the initial financial cost of your dental treatment?
8. In hindsight, would you undergo the treatment you had for your mouth and teeth again?

Each patient was asked to fill out the questionnaire themselves to ensure as little bias as possible.

### 2.5. Statistical Analysis

Two separate investigators (P.D., E.R.) extracted the required data from the questionnaire and inserted it into two separate spreadsheets, which were then compared to check for any discrepancies; a single database was then obtained, which included the participants' demographic data, their responses to the questionnaire, and the clinical characteristics previously recorded.

Descriptive statistics were used to illustrate the overall gathered responses as means and standard deviations (SD), while binary outcomes were reported as a prevalence.

The outcomes of interest were the patients' responses to the questionnaire, recorded on a 1–10 VAS scale (continuous variable), and the willingness to undergo the procedure again if needed (binary outcome, yes/no).

Linear (for continuous variables) and logistic (for binary outcomes) regression models were produced depending on the stated outcomes to test the correlations between the gathered patient responses, demographics, and obtained clinical data to the stated variables of interest. A stepwise regression approach was used for the variables of interest to test their predictive values, and the variables were kept for multivariate modeling if they obtained *p* < 0.05.

All analyses were performed in R Studio (Integrated Development for R. RStudio, PBC, Boston) by a separate investigator.

## 3. Results

Table 1 presents the characteristics of the sample, divided according to the design of the rehabilitations.

Among the 71 patients eligible for recruitment, 48 were contacted and agreed to participate in the present evaluation. Among them, eight (16.6%) were excluded because they were unable to remember the location of the procedure. Therefore, 40 patients constituted the sample in the present study.

Twenty-seven patients received monolithic zirconia restorations, while 13 received partially veneered zirconia restorations; 26 patients received a FPD, while the remaining 14 received a SC. 27 patients were treated with a conventional workflow, while 13 with a digital workflow.

The success rate for monolithic restorations was 92.6%, while that for the veneered restorations was 92.3%. Overall, two restorations (5%) had a mechanical complication: one screw-retained monolithic single crown experienced a screw loosening, which was solved by retightening the screw at 35 N/cm, and one 4-unit, cement-retained, veneered FPD, occluding with a fixed partial denture, underwent a chipping of the veneering ceramic, possibly as a consequence of the parafunctional behavior of the subject; as the chipping was minor, this complication was resolved only by polishing the surface.

**Figure 1:**
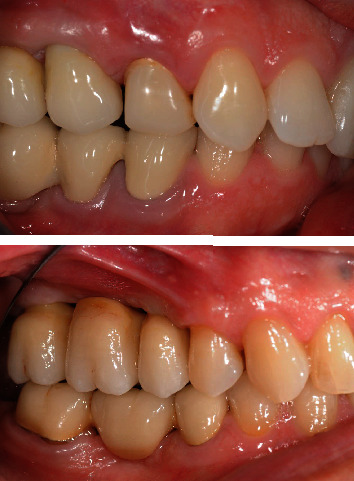
Peri-implant mucositis of the 1.5 and peri-implantitis of the 1.7.

Three implants (4.2%) experienced biological complications: one implant supporting a FPD had signs of peri-implantitis, while two other cases of FPD had signs of peri-implant mucositis (Figure 1). At the recall appointment, patients with biological complications underwent a motivational session and a session to reexplain oral hygiene instructions followed by a professional nonsurgical therapy together with the use of chlorhexidine mouthwashes and gels.

The survival rate was 100% for both the monolithic restorations and partially veneered restorations, as all implant-supported restorations were still functioning at the 3-year mark, irrespective of the condition of the implants or the restoration.

At the 3-year follow-up, medical loupes and 5x magnification revealed no wear of the restorations and the opposing dentition.

### 3.1. Patients' Responses and Correlation to Recorded Outcomes

**Figure 2:**
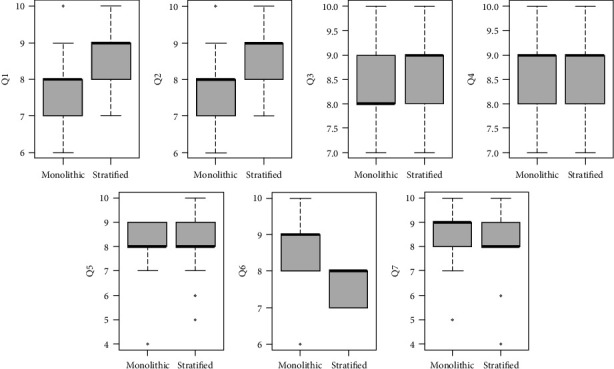
Box plots of the recorded questionnaire responses.

Figure 2 presents the responses to the questionnaire. Patients that had a partially veneered restoration believed their restoration to be more aesthetically pleasant both when it was delivered (Q1) (0.76, *p* < 0.05) and as it appeared at the recall appointment (Q2) (0.85, *p* < 0.01) than those who received a monolithic restoration. The other measured outcomes had no effect. Patients who had >2 implants (-0.9, *p* < 0.05) or who had tilted implants (-0.58, *p* < 0.05) reported more difficulties in maintaining proper oral hygiene (Q5). Moreover, patients who experienced biological complications were more likely to report higher difficulties in maintaining proper oral hygiene (Kruskal-Wallis, *p* < 0.01).

Patients that received partially veneered restorations reported being much less happy about the price of their restoration (Q6) when the prosthesis was delivered (-0.94, *p* < 0.001), but this difference was no longer definable at the last recall appointment, as patients who received screw-retained restorations (1.76, *p* < 0.001) were more likely to report a high score to Q7, while patients who experienced complications (-2.5, *p* < 0.001) or patients with a single implant restoration (-1.14, *p* < 0.05) were less satisfied with the payment they had made years before. None of the recorded factors played a role in determining the perceived capability to speak (Q3) or chew (Q4).

### 3.2. Willingness to Undergo the Same Procedure

Overall, the procedure was fairly acceptable to patients, as only three were not willing to undergo the same procedure (8%). Patients who had a complication, either biological or mechanical, were much less likely to be willing to repeat the same procedure (odds ratio [OR] 22.72, *p* < 0.05); no other correlations were found with the recorded outcomes.

## 4. Discussion

The main purpose of the study was to focus on patient perceptions of implant-supported rehabilitations performed using monolithic or partially veneered zirconia. CAD/CAM procedures and digital workflow are now common in daily clinical practice because of their excellent results in terms of use and quality obtained [21].

The secondary purpose was to assess the success and survival rate of monolithic or partially veneered zirconia restorations after 3 years. In the present study, we included patients treated in two clinical settings by two experienced prosthodontists following standardized clinical protocols. All parameters investigating the patient's perceptions were assessed using a questionnaire administered by the same investigator to achieve objective results. Furthermore, to include only patients that had actual recollection of the procedure, we excluded patients that did not remember the side of the restoration.

The literature has an increasing number of clinical long-term studies of implant-supported restorations, with relevant information on the clinical outcomes. However, patient-evaluated dentistry is increasingly being recognized as a necessary consideration to determine the overall prosthodontic success. Information on patient satisfaction following the clinical protocols described in the present study are still lacking in the literature [20].

The results of our investigation confirm that, after a 3-year follow-up, monolithic and partially veneered zirconia restorations are both well-accepted treatment options. The results also show that partially veneered restorations are associated with a statistically significant higher aesthetic score, outlining the fact that patients find veneered restorations more aesthetically pleasant than monolithic restorations, but in our sample, no monolithic restorations experienced any fracture, while one veneered crown experienced chipping of the veneering material.

However, favourable aesthetic results were achieved for both groups. On the other hand, patients perceived no differences from a functional point of view, considering the reported ability to chew and speak, similar to that reported in the literature [22].

The analysis of the questionnaire indicated that patients who had >2 implants or who had tilted implants reported more difficulties in maintaining proper oral hygiene. Furthermore, patients who developed biological complications reported having had difficulties in maintaining proper oral hygiene. This finding is in line with the results of Pons et al. [23], who observed that poor access to proximal hygiene presented increased risk of developing peri-implant disease, in particular peri-implant mucositis. Furthermore, the same study also reported that the presence of peri-implant disease was related to self-reported assessment of oral hygiene measures and to patient perception of gingival/mucosal bleeding when performing oral hygiene.

These results show the ability of the patients, who were carefully selected and enrolled in a maintenance program, to perceive when proper oral hygiene was performed. Finally, the significant association observed between the occurrence of complications and the willingness to undergo the same procedure or the cost perceived by the patients should also be considered with care. Gargallo-Albiol et al. reported similar results on patients' perception of dental implant removal following complications, reporting a certain reluctance in patients to undergo future implant placement in the same clinic or with the same professional [24].

Our report also states that monolithic zirconia crowns have good clinical performances, given the low complication rate reported. This is the major advantage of monolithic zirconia crowns over conventional veneered restorations: for the latter, even when zirconia-based, chipping of the veneering material is the main and most frequent technical complication [25], whereas Y-TZP is the toughest ceramic material available on the market, notably reducing the rate of complications for the former [26]. In the present paper, monolithic restorations had a higher success rate than veneered restorations, similarly to that reported by other authors [27, 28].

Although HT zirconia is much more resistant than porcelain, vestibular feldspathic veneers are still preferred in cases where aesthetic is paramount, such as the restoration of a central incisor, or when the chromatic characteristics of the adjacent teeth make it difficult for the dental technician to properly give the crown the required color. According to our findings, monolithic zirconia was aesthetically satisfactory and accepted by the patients, even after 3 years.

Some papers have reported the possibility that the techniques used to superficially color zirconia may not be stable over time [29, 30], as this superficial layer could potentially wear off as a consequence of the natural abrasions that occur during use. We did not report this phenomenon in the present study, as the monolithic zirconia crowns were all integrated in the oral cavity and the patients reported no change in their color, similarly to what other papers have found [31, 32].

Moreover, eliminating the necessity of a veneer enables the restoration to be thinner; the preparation on natural teeth can be less extensive as a monolithic zirconia crown with a thickness of 0.7 mm on the occlusal surface has sufficient fracture resistance to be used as an implant-supported restoration [33].

One previously reported flaw of monolithic zirconia crowns is that, given the superior hardness of the material (Hv ≈ 1200 GPa; double that of porcelain [34]), zirconia could be more abrasive to enamel than other restorative materials, at least on paper, especially if not polished properly [35]; recent systematic reviews have concluded though that monolithic zirconia is not more abrasive than other, commonly used, restorative materials, at least in in vitro studies [36, 37].

From our clinical observations, wear of enamel on the antagonist teeth of monolithic zirconia crowns was no different from that observed on the adjacent teeth.

Implant-supported SCs are the standard of care for replacing a missing single tooth, and implant-supported monolithic zirconia crowns have a high survival rate, comparable to classical PFM (porcelain-fused-to-metal) crowns [38]. They can be both cement-retained or screw-retained, and while both are clinically acceptable, screw-retained crowns have some clear advantages and are therefore nowadays preferred, as extrusion of cement in the peri-implant tissues during cementation can lead to biological complications [39]. Also, when monolithic zirconia crowns are fabricated to be hybrid cement-screw-retained, they are cemented extraorally on titanium bases; the abutment is completely surrounded by zirconia, thereby having an aesthetic material underneath the soft tissues, avoiding the greyish aspect that sometimes can develop, especially in extremely thin biotypes, with restorations cemented on titanium abutments [40].

The main limitation of this study is its retrospective nature, as patients that did not agree to the recall visit might have had a higher rate of complications, which barred them from attending the appointment.

Moreover, our sample is quite heterogenous, as we included both SCs and FPDs on two or three implants, axial and tilted. Finally, our considerations on color and wear of the delivered prostheses are only based on our clinical observations, as no volumetric or colorimetric approach was adopted.

Long-term randomized controlled studies should be carried out to determine the patient perception of these two prosthetic approaches and their clinical reliability in standardized clinical conditions.

## 5. Conclusions

Given the high success rate found in the present study, monolithic and partially veneered zirconia restorations can both be defined as reliable treatment options for implant-supported SCs and FPDs. However, a statistically significant difference was found, outlining the fact that veneered restorations are more esthetically pleasant than monolithic restorations. Therefore, especially in the visible area, adding a partial veneer can improve the aesthetics while at the same time maintaining an acceptable rate of complications.

## Figures and Tables

**Table 1:** Characteristics of the sample.

Characteristic	Monolithic	Partially veneered
*Number of patients*	27 (67.5%)	13 (32.5%)
*Type of rehabilitation*		
Fixed partial denture	17	9
Single crown	10	4
*Type of prosthesis*		
Cement-retained	6	5
Screw-retained	21	8
*Parafunction*		
Yes	5	5
No	20	8
*Number of implants*		
1	10	4
2	14	7
3	3	2
*Number of mechanical complications*	1 (3.7%)	1 (7.7%)
*Number of biological complications*	2 (7.4%)	1 (7.7%)
*Would repeat the treatment?*		
Yes	26	11
No	1	2

## Data Availability

The data of the study can be asked to the corresponding author.
